# Biochar and Dairy Manure Amendment Effects on *Cynodon dactylon* Performance and Soil Properties

**DOI:** 10.3390/plants13020242

**Published:** 2024-01-15

**Authors:** Lisandro J. Entio, Cosette B. Taggart, James P. Muir, Eunsung Kan, Jeff A. Brady, Olabiyi Obayomi

**Affiliations:** 1 Texas A&M AgriLife Research and Extension Center at Stephenville, 1229 US-281, Stephenville, TX 76401, USA; cosette.taggart@go.tarleton.edu (C.B.T.); eunsung.kan@ag.tamu.edu (E.K.); jeff.brady@ag.tamu.edu (J.A.B.); biyi.obayomi@ag.tamu.edu (O.O.); 2 Wildlife and Natural Resources Department, Tarleton State University, P.O. Box T-0050, Stephenville, TX 76401, USA

**Keywords:** dairy manure, biochar, soil nutrients, *Cynodon dactylon*

## Abstract

Studies have determined the separate effects of biochar (BC) and manure application on forage species and soil, but few examined the effects of BCs made from different feedstock applied along with dairy manure. We compared the effect of wood- and manure-derived feedstock BC as well as dairy manure amendment application on *Cynodon dactylon* performance and soil properties in sandy loam and clay loam soils in a greenhouse pot study. Plant samples were assayed for herbage and root dry weight as well as herbage and root N and C percent and yield. Soil samples were assayed for macronutrients, micronutrients, metals, pH and conductivity. Data analyses involved variance analysis and Tukey’s tests using R in RStudio (the IDE). In general, *C. dactylon* yields or mineral content were not affected by either manure or BC. However, an increase in the total herbage dry weight (30%) and in herbage N% (55%) was observed for clay loam and sandy loam soil, respectively, due to manure amendment application. There were no alterations in clay loam NO_3_-N and P due to any treatment; however, in sandy loam, these nutrients were not altered only when wood BC was applied. In sandy loam soil, NO_3_-N and P increased when manure BC along with dairy manure and when manure BC alone were applied, respectively. Thus, wood BC application should be considered to avoid these nutrient buildups when dairy manure is used as a soil amendment. This research shows a neutral (BC) or positive (dairy manure amendment) impact on *C. dactylon* performance. BC incorporation increases soil total C, showing potential for C sequestration. Long-term field trials could corroborate plant performance and soil parameters.

## 1. Introduction

Manures and compost in agricultural production have been used at least since the 3rd millennium BC in the Middle East [1]; biochar (BC) is also an ancient technology for improving soil fertility for agriculture dating from 8000 years ago in South America [2]. Currently, around the world, the amendment of soils using cow manure (mix of urine, feces, waste food, bedding material and straw) for supplying plant nutrients is a very common practice [3]. However, the high content of nutrients often results in losses (e.g., leaching or volatization) of those not immediately taken up by crops during the growing season [4]. 

In the United States of America, each one of the 9.38 million dairy cows [5] can produce up to 13.5 Mg/year of wet manure (11 kg P/year) [6]. The application of this manure as an organic fertilizer (i.e., solid or liquid/slurry amendment) in general is based on N-targeted crop needs, leading to P [7] and pathogen [8] oversaturation and increasing runoff risk with consequences such as eutrophication and surface water contamination [7]. 

One alternative to mitigate these problems could be combined organic fertilizer (i.e., manure) with BC (porous carbon material produced by pyrolysis of organic wastes including animal manure, crop residues, grass and forest waste [8]). It adsorbs nutrients and organic matter from soils, whether added as manure or fertilizer [9], as well as from anaerobic digestates [10] and livestock wastewater [11], offering the possibility of decreasing environmental nutrient pollution [8]. At the same time, BC can be an adsorbant for pesticides [12], antibiotics [13,14] and metals [15]. Moreover, as it is a more stable source of soil C than manure, it is emerging as a possible strategy for carbon sequestration [16]. 

Biochar as a soil amendment to improve soil fertility and row crop yields has been well documented as it improves soil moisture and nutrient retention as well as soil microbiome health [17]. Several studies, mainly in annual row crops, reported that biochar increases plant productivity, with an average yield increase of 10–42% [18,19,20]; however, short-term (first growing season) biochar amendment efficacy for ameliorating pasture soil characteristics and increasing forage yields and nutritive values in warm climates has not been widely tested and remains controversial. In temperate climates, research results indicate that initial soil fertility effects can be negative when they bind nutrients that then become unavailable to forage root uptake. Its eventual effects on subtropical herbage characteristics are particularly lacking.

Research on BC and manure amendment application to forage systems in different soils indicates effects on plant productivity as well as on soil parameters that vary with crop production system (i.e., species, tillage, rotation, etc.) and soil type (i.e., textures), especially when both are applied simultaneously. However, very little research exists for the combined effects of manure and BC obtained from diferent feedstocks on perennial, subtropical forages. We chose *Cynodon dactylon* because of its widespread distribution in the subtropics, including regions with low rainfall, extreme temperatures and soils deficient in key macronutrients and micronutrients. It also was chosen because it is a forage crop which may be utilized in a forage system designed for nutrient removal [21].

Artiola et al. [22] and Niraula et al. [23] reported a positive effect on *C. dactylon* growth when wood BC was applied in sandy loam soil and loamy sand soil, respectively, whereas Nystrom [24] reported a neutral effect when sorghum-derived BC was applied both in a greenhouse experiment with different soil textures and in a field trial in a sandy loam soil. Manure amendment had a positive effect not only on *C. dactylon* [25,26] but also on *Festuca arundinacea* and *Digitaria erianta* [27] forage yield in sandy loam and sandy clay loam soil, respectively. Other studies of manure and BC in different forage species and soils reported a neutral effect on *C. dactylon* performance [28] and a positive effect on crops like radish [29] and cotton [4]. Several studies looking at soil parameters reported a wide range of contradicting BC effects [30,31,32,33,34,35]. 

Studies have determined the separate effects of BC and manure amendment application on *C. dactylon* and other forage species’ performance and soil characteristics, but few examined the effects of BCs made from different feedstock on plant growth and soil parameters in combination with manure amendment application. Our goal was to explore new strategies to dispose of dairy wastes and at the same time recycle nutrients on farms or at a regional scale with no negative impacts on *C. dactylon* production systems. We hypothesized that the application of manure BC would have fewer detrimental effects compared to wood BC when applied along with dairy manure. Specifically, we examined *C. dactylon* production and nutrient content as well as sandy and clay loam soil nutrient levels. In our study, the objective was to compare the effect of wood- versus manure-derived BC and manure amendment application on *C. dactylon* herbage and root yield and mineral content as well as soil properties in sandy versus clay loam. 

## 2. Materials and Methods

### 2.1. Experimental Design

This greenhouse study was conducted at the Texas A&M AgriLife Center in Stephenville, TX, USA (32.2454° N, −98.1970° W) over a 90-day period. Each pot was considered an experimental unit, and all treatment combinations were replicated three times. This was essentially two parallel (soil types) three-factorial experiments: (1) BC type; (2) BC application; and (3) manure application. Soil was not considered a factor because initial analyses of variance indicated that each soil responded to the other factors in very distinct ways. Therefore, identical studies were carried out simultaneously on sandy and clay loam. 

### 2.2. Soil Preparation

Soil was collected from the top 20 cm of a Windthorst fine sandy loam and a clay loam in Stephenville, TX, USA. It was homogenized, air-dried under ambient conditions, sifted and distributed in 3 kg units to 96 4-L plastic nursery pots. A sandy loam was selected because it is a common texture in this region and BC amendments tend to be more effective in course-textured soils [36]. Also, a clay loam was selected to test a soil with a heavier texture. 

### 2.3. Manure

Dry dairy manure obtained from Tarleton State University Dairy (10 Mg DM/ha) was compared to a no-manure control (Table 1). Manure was screened from dairy stall flush spillways for collection; sand was removed and then left to dry in the sun for two weeks before application. Manure application was based on grass P requirements rather than N. Sanderson and Jones (1997) [37] reported that annual compost application rates for manure as fertilizer should not exceed 44.8 Mg/ha in the southern USA when applied to *C. dactylon* cv. Coastal. This maximum rate was based on the average amount of P in the manure and forage requirement and is recommended to prevent soil P accumulation and NO_3_-N leaching in soil. In our study, a 10 Mg manure DM/ha rate was used because it met forage P requirements and equated to 45 Mg/ha before drying.

### 2.4. Biochar

BC originating from manure (Ecochar, Evansville, IN, USA) and wood (Waste to Energy, Inc., South Slocomb, AL, USA) (Table 2) was compared to a no-BC control. Physiochemical characteristics are described in Table 1. BC was ground using a Thomas Wiley Mill (Swedesboro, NJ, USA) fitted with a 2 mm screen. BCs were incorporated in pots, replacing 2% of soil on a dry matter weight percentage (i.e., 60 g BC/2940 g of soil).

### 2.5. Treatments

Each experiment included three factors: BC source (manure vs. wood), BC application (+ or −) and manure application (+ or −). Twelve distinct treatment combinations (Table 3) resulted: (1) soil (control without forage); (2) soil + forage (control with forage); (3) 2% wood BC; (4) 2% wood BC + forage; (5) 2% wood BC + 10 Mg DM/ha manure; (6) 2% wood BC + 10 Mg DM/ha manure + forage; (7) 2% manure BC; (8) 2% manure BC + forage; (9) 2% manure BC + 10 Mg DM/ha manure; (10) 2% manure BC + 10 Mg DM/ha manure + forage; (11) 10 Mg DM/ha manure; (12) 10 Mg DM/ha manure + forage. 

### 2.6. Seeding and Watering

Because *C. dactylon* propagates vegetatively, it was pre-cultured before the experiment, and a 15 cm sprig was transplanted into each pot. Pots were watered as needed (~5–7 days) to maintain near-field capacity, and leachate was recycled back into the soil. The experiment was conducted in the greenhouse for 90 days. All treatment combinations were applied in triplicate pots constituting three blocks, which were tables in the greenhouse.

### 2.7. Sampling and Sample Preparation

#### 2.7.1. Soil

At the termination of the trial, soil sub-samples representing 0.5% of total pot soil were taken from each experimental unit (pot) using a small soil probe to minimize root loss and account for a complete cross-section of soil. The samples were allowed to air-dry under ambient conditions until weight stabilized, then they were sifted.

#### 2.7.2. Forage

Plants were sheared at soil level two times after planting. After the second harvest, roots were washed with water to remove all remaining soil. All samples were dried in a forced-air oven at 55 °C until weight stabilized. Biomass was recorded immediately after removal from the oven. All samples were ground though a 1171H10 Wiley Mill (Thomas Scientific, Swedesboro, NJ, USA) fitted with a 1 mm screen.

### 2.8. Sample Analysis

Soil samples were assayed for the determination of permanganate oxidizable carbon using a method adapted by Culman et al. [38]. In addition, total carbon percentage was determined. Soil samples were additionally assayed by Texas A&M AgriLife Extension Service—Soil, Water, and Forage Testing Laboratory using extractants described by Mehlich [39]. Data received from this lab included pH, conductivity, P, K, Ca, Mg, S, Na, Fe, Zn, Mn and Cu values. Additionally, soil NO_3_-N data were provided using a Cd reduction [40,41]. Total volatilizable N and C percentages were determined by combustion in a Leco CN828 (Leco Corporation, St. Joseph, MI, USA).

Plant samples were assayed for volatilizable C and N content via a Leco CN828 elemental analysis by combustion. For herbage and root samples, the percentages given by the assay were multiplied by the total weight of samples to determine total weight of C and N in grams.

### 2.9. Statistical Analysis

Data were analyzed using R (R-4.2.2) (R Core Team, 2022). Independent variables consisted of BC type, BC application and forage inclusion. Dependent variables consisted of soil and plant-captured N, P and C content, as well as dry weight, C and N herbage and root yields. Data collected were normally distributed and showed variance homogeneity so parametric data analyses were used. A Tukey’s test was used to test for significant (*p* ≤ 0.05) differences among factors for simple effects of dependent variables grouped by treatment only if factors did not interact. We considered significance at *p* ≤ 0.05 and did not report individual probabilities in the text unless they were *p* > 0.05 and relevant to the discussion. Also, Pearson’s correlation analyses and multiple regression analyses (forward) were performed to find associations and the cause–effect relationship among plant and soil parameters.

## 3. Results

### 3.1. Plant Performance

In the sandy loam, neither *C. dactylon* yields nor mineral content were affected by any treatment or combination of them. An exception was N% at the second herbage cut (N% H-DW2), which was affected by the main factor “manure application”. Thus, the application of manure amendment increased N plant content by 55% compared with the treatment without manure (Table 4). Similarly, in the clay loam soil, no treatment nor combination changed *C. dactylon* herbage and root yield or nutritive value; however, total herbage dry weight (TH-DW) was an exception affected by the main factor “manure application”. Thus, the application of manure amendment increased total herbage yield by 30% compared with pots without manure (Table 4).

### 3.2. Soil Parameters

#### 3.2.1. Macronutrients

Total C in the sandy loam was affected only by the main factor “BC application”. Thus, when BC was applied, total C increased by 34% compared with the control (Table 5). In the case of clay loam soil, total C was affected by the main factors “BC application” and “Manure application”. Thus, total C level increased by 24% and 11% when BC and manure were applied, respectively (Table 5). Oxidizable C was affected only in the sandy soil by the main factor “Manure application”. Thus, the application of manure increased oxidizable C content by 86% compared with the treatment without manure (Table 5). Total N in the sandy loam was not affected by any treatment or combination, whereas NO_3_-N was affected by the main factor “plant application” and the two-factor interaction “BC type × manure application”. The inclusion of *C. dactylon* reduced NO_3_-N soil content by 94% compared with the bare soil (Table 5). With respect to the two-factor interaction “BC type × manure application”, only manure BC in combination with manure amendment application differed (312%) from its treatment without manure application (Table 6a). In the clay loam, only the total N was affected by the main factor “Manure application” and the three-factor interaction “BC type x BC application x Plant application”. Thus, when manure was applied, total N level increased by 17% (Table 5). For the “BC type x BC application x Plant application” interaction, only manure BC + *C. dactylon* differed from control pots, showing a 65% total N increase (Table 7). 

P was affected only in the sandy loam soil by the main factor “Plant application” and the three-factor interaction “BC type × BC application × Manure application”. The inclusion of *C. dactylon* reduced P soil content by 24% compared with the bare soil (Table 5). With respect to three-factor interaction “BC type × BC application × manure application”, P soil content only increased (average 262%) when manure BC was applied, regardless of whether it was combined or not with manure amendment. Wood BC application showed no difference compared with the control, regardless of whether it was combined with manure amendment or not (Table 8).

K in the sandy loam was affected by the two-factor interactions “BC type × BC application” and “BC application × plant application”. Regarding the “BC type × BC application” interaction, only manure BC showed differences, with the control increasing soil K content by 425% (Table 6b). With respect to the “BC application × plant application” interaction, with or without plant application, when BC was applied, K soil content increased by 279 and 174%, respectively; however, regardless of whether BC was applied or not, plant presence differently decreased K soil content by 19 and 42%, respectively (Table 6c). In the case of clay loam soil, K was affected by the two-factor interaction “BC application × Plant application”. Without BC application, the inclusion of *C. dactylon* decreased soil K content by 49%; however, when BC was applied, plant application did not reduce K soil content (Table 9).

Na in the sandy loam was affected by the main factor “manure application” and the two-factor interaction “BC type × BC application”. The application of manure increased Na content by 31% compared with the treatment without manure (Table 5). Regarding the two-factor interaction “BC type × BC application”, only the application of manure BC showed differences to the control by increasing Na soil content by 197% (Table 6b). In the case of clay loam soil, Na was affected by the three-factor interaction “BC type × BC application × manure application”. Na soil content increased by 126% when manure BC combined with manure amendment was applied. Wood BC application showed no difference to the control, regardless of whether it was combined with manure amendment or not (Table 10). Ca was not affected by any main factor or interaction in either soil. 

#### 3.2.2. Micronutrients

Mg was affected only in the sandy loam soil by the main factor “manure application” and by the two-factor interaction “BC type × BC application”. Manure amendment increased Mg content by 20% compared with the treatment without manure (Table 11). With respect to the two-factor interaction “BC type × BC application”, only the application of manure BC showed differences to the control, increasing Mg soil content by 61% (Table 12). Similarly, Mn was affected only in the sandy loam by the main factor “plant application”. Thus, *C. dactylon* inclusion decreased Mn content by 14% compared with the bare soil (Table 11). S was not affected by any main factor or interactions in the sandy loam; however, it was affected by the main factor “plant application” in the clay loam soil. Thus, *C. dactylon* inclusion decreased soil S content by 27% when compared with the bare soil treatment (Table 11). 

#### 3.2.3. Metals

In the sandy loam, Cu was affected by the main factor “manure application”. Manure amendment application decreased soil Cu content by 24% compared with the treatment without manure amendment (Table 13). Fe was affected by the main factors “plant application,” “manure application” and “BC application”. The inclusion of *C. dactylon* showed 27% more Fe than bare soil treatment, whereas manure amendment application, as well as BC application, showed 23% less Fe than their respective controls (Table 13). Zn was not affected by any main factors or interaction of them.

In the case of clay loam soil, none of the analyzed metals was affected by any main factor or interaction.

#### 3.2.4. pH and Conductivity

Sandy loam pH was affected by the main factors “plant Application” and “manure application” as well as by the two-factor interaction “BC type × BC application”. The inclusion of *C. dactylon* resulted in a 1% greater pH than the bare soil, whereas the application of manure resulted in a 1% lower pH than the treatment without manure (Table 14). The two-factor interaction “BC type × BC application” indicated that the application of manure BC differed to the control, increasing pH soil content by 9% (Table 15). In the case of the clay loam soil, pH was affected by the three-factor interaction “BC type × BC application × manure application”. pH showed an increase (3%) only when manure BC (without manure amendment) was applied. Wood BC application showed no difference to the control, regardless of whether it was combined with manure amendment or not (Table 16).

Conductivity in the sandy loam soil was affected by the main factors “plant application” and “manure application”, and by the two-factor interaction “BC type × BC application”. The inclusion of *C. dactylon* resulted in 27% less conductivity than the bare soil, and the application of manure increased conductivity by 34% compared to without manure (Table 14). The two-factor interaction “BC type × BC application” indicated that the application of manure BC increased soil conductivity by 94% (Table 13). In the case of clay loam soil, it was affected by the two-factor interaction “BC application × Plant application”. Only without BC application did the inclusion of *C. dactylon* affect conductivity (−61%); however, when BC was applied, plant application did not affect soil conductivity (Table 17).

## 4. Discussion

### 4.1. Plant Parameters

In general, considering both tested soils, neither herbage nor root yield nor herbage nutritive value of *C. dactylon* parameters was affected by any treatment or combination. However, there was one exception for each soil type. An increase in the total herbage dry weight (30%) and in N% at the second herbage cut (55%) was observed for clay loam and sandy loam, respectively, due to manure amendment application.

The increase in *C. dactylon* total herbage dry weight only in the clay loam soil may be a result of it being a soil that is richer in nutrients with a higher conductivity compared with sandy loam soil. This characteristic could have contributed to mitigating the first short-term stage effects of BC as a sink for soil nutrients competing with root–plant nutrient uptake due to its porosity and high specific surface area [9]. In general, the lack of short-term BC effect might be explained because most nutrients in the amendments are in organic form in BC so gradual microorganism degradation is needed for plant-available nutrient release, slower than that of organic fertilizer availability [4].

These results contradicted the greenhouse pot trials of Artiola et al. [22] and Niraula et al. [23], in which there was a positive effect on *C. dactylon* growth when wood BC was applied in sandy loam soil and loamy sand soil substrate, respectively. The absence of BC effect found in our research on *C. dactylon* performance agreed with Nystrom’s [24] results from a greenhouse trial which included sandy and clayed soil textures as substrates; however, they only partially agreed with Niraula et al. [23], who also reported a neutral response when intermediate loading rates of wood BC were applied but a positive (saturated BC at all loading rates and unsaturated BC at the highest loading rates) and negative (unsaturated at low loading rates) effect when wood BC was applied to pots with sandy loam soil substrate. Nystrom [24] also reported no BC effect on *C. dactylon* performance in a field trial carried out in sandy loam soil.

Other authors reported a greater effect of raw and composted [25] and composted [26] dairy manure when compared with unamended/unfertilized or with/without supplemental inorganic N treatments, respectively, on *C. dactylon* forage yield growing in sandy loam soil. The same positive effect was reported for *Festuca arundinacea* and *Digitaria erianta* growing in sandy clay loam [27]. However, neither of the previously mentioned studies evaluated BC effects in combination with manure amendment application or vice versa. Cooper [28] partially agreed with our results, reporting no wood BC nor dairy manure amendment effect on *C. dactylon* field production or nutritive value across three different soils (i.e., sandy, sandy loam, clay loam). In another field trial where the combined effect of poultry manure and wood BC was tested for radishes growing in a sandy loam soil, a positive yield effect of BC alone appeared only in the second year, while a plant yield increase due to the combination of BC and poultry manure was measured in the first year [29]. The application of organic amendment in combination with BC in our results did not with agree with Zhang et al. [4] since they reported a greater effect on cotton yield when BC (i.e., cotton straw) was applied along with poultry litter than when litter was applied alone. 

### 4.2. Soil Parameters

#### 4.2.1. Macronutrients

In general, most of the analyzed macronutrients were altered by soil amendments in the sandy loam, whereas, in the clay loam soil, only two were altered. For both studied soils, total soil C content increased as BC load increased. This result agreed with the tendency reported by Demisie et al. [42] when wood or bamboo BC was applied in a clay loam soil, although they did not evaluate any manure amendment. In our research, clay loam soil had an increase in total C content when dairy manure was applied alone. These results agreed with a meta-analysis which showed that clay-texture soils had greater soil C pool increase rates compared to sandy textures soils when manure amendment was applied [34]. This increase was expected since the BCs we studied had at least 23% fixed C.

Soil oxidizable C was not affected by BC application in sandy loam but increased when manure amendment was applied. These results agreed with other studies where no changes in oxidizable C content were reported in sandy loam soil when similar BC derived from wood or manure was tested in *T. incarnatum* and *L. multiflorum.* However, no manure treatments were tested in those studies [36]. It is worth noting that, in case of the sandy loam, regardless of BC application, the incorporation of dairy manure amendment by itself increased oxidizable C soil levels by 86% in our study compared with the no-manure treatment. The fact that we found alterations in oxidizable C only for the sandy soil may be expected because it initially contained approximately only 24% of the oxidizable C found initially in the clay loam soil. 

Total N was not affected by any factor or combination in the sandy loam soil, while, in the clay loam soil, a slight increase was observed due to the application of manure. This result agreed with Anger and N’Dayegamiye [43], who also reported a total N increase when cattle manure was applied in silt loam soil. NO_3_-N was not affected by any factor or combination in the clay loam soil. However, in the sandy loam soil, the application of any of the BCs alone tended to reduce the original NO_3_-N soil content, indicating its capacity for adsorbing some nutrients on its surface [44] and making them available for an extended period [45]. Also, the presence of *C. dactylon*, regardless of any other factor, showed a high reduction (94.1%) in NO_3_-N soil content due to its own uptake for growing and because of the conversion to unavailable forms. On the other hand, the incorporation of manure BC along with dairy manure tended to increase the NO_3_-N level when compared with control pots with no manure amendment. This could be attributed to the ability of BC to increase soil N retention [46] and maybe manure nutrient utilization efficiency [29]. In our study, BC alone did not increase N soil level, contradicting the reported general tendency that BC treatments increase soil N content [47]. Other authors also reported the same tendency when saturated or unsaturated wood BC was applied in an experiment carried out with the same grass species and soil [23] as ours. 

P was not affected by any factor or combination in the clay loam soil. Although sandy loam P was reduced by the presence of *C. dactylon* (24%) by itself, the application of manure BC increased P soil content (average 261.5%), regardless of manure amendment application, whereas wood BC did not alter P soil content independently whether manure amendment was applied or not. That we found considerable alterations in P only for the sandy soil may be expected because it initially contained only 10.8% of the P found in the clay loam soil. These results agreed with a study which reported similar effects of manure and wood BC on soil P content [36]. Moreover, our results did not agree with a study which reported that P content increased when wood BC was applied in an experiment where *C. dactylon* was grown in a sandy loam [23]. If P binds to BC particles, the P increase detected could be positive in the long term for soils, water health and plant growth. On the other hand, because BC adsorption is linked to P concentration ([P]) (at high [P], the P sorption rate slows due to competition for binding sites) [48], excess P in soils might increase nutrient runoff with negative impacts on non-targeted downstream ecosystems like ground and surface water sources [7]. Regarding N and P alterations in sandy soils due to BC and/or organic amendment application, neither of them alone increased these nutrients’ availability when applied in a peanut–wheat rotation, but there was synergistic effect when they were combined [31]. 

K was altered by BC application in both studied soils. In general, K increased by 174 to 425% in sandy loam soil when BC was applied, showing the highest increase percentage with the incorporation of manure BC. This result agreed with Nystrom [24], who reported a soil K increase when *C. dactylon* was amended with BC in a sandy loam field trial, and with El-Shony et al. [31], who reported the same effect in a peanut–wheat rotation in a sandy soil. Major et al. [30] reported the same effect in a maize–soybean rotation in an oxisol. BC application tended to maintain clay loam K soil level, compensating *C. dactylon* uptake as well as possible leaching and/or immobilization. The considerable alterations in K only for the sandy soil may be expected because it first contained around 12.5% of the K found in the clay loam soil. Both soils already had the critical K level (165 ppm) to avoid *C. dactylon* yield losses [24]. 

In the case of Na, both soils showed alteration due to BC and/or manure application. Thus, a Na increase was observed in sandy loam soils when either manure BC or manure amendment was incorporated alone. In the clay loam soil, Na increased only when both amendments were incorporated simultaneously. This may be expected since sandy loam control pot Na levels showed only 25% of the Na found in clay loam; therefore, a higher input of this cation would be necessary to alter its content in clay loam soils. Finally, Ca soil level was not affected by BC or manure amendment application in either of the soils. This last result did not agree with Major et al. [30], who reported a Ca level increase when wood BC was applied in a maize–soybean rotation.

#### 4.2.2. Micronutrients

Only Mg in sandy loam soil was altered (increased) by BC or manure amendment alone. By contrast, Mn and S were only affected (decreased) by *C. dactylon* inclusion in sandy loam and clay loam soil, respectively. In this sense, our results showed that, in the sandy loam soil, the incorporation of manure BC alone increased Mg three times more than when dairy manure amendment was applied. This might be expected since the applied manure BC had a higher content of Mg than the dairy manure amendment. It might be because the application of alkaline BC may reduce soil acidity, thereby increasing soil pH and thereby enhancing the availability of alkaline cations (e.g., Mg^2+^) [30,49,50]. However, it would not seem to be the cause in this study because the BC was not sufficiently alkaline (pH 8.8).

#### 4.2.3. Metals

Two out of the three analyzed metals (i.e., Cu, Fe) were altered in the sandy loam, whereas none was affected in the clay loam soil. Cu and Fe decreased when dairy manure was applied alone and when dairy manure or BC was applied alone, respectively. In the case of Fe, it also decreased when *C. dactylon* was included. Cu and Fe content was more than two and four times greater, respectively, in the clay loam than in the sandy loam soil. In our short-term study, no limiting or toxic effect of soil metal content on the studied forage grass species was detected. Moreover, the application of manure amendment reduced Cu and Fe content. This effect was also reported by Kusiemska et al. [32] for Cu when cattle manure was applied as an amendment in a 2-year pot experiment in a sandy soil. This might be because the organic component in manure by-products has a high affinity for metal cations because of the presence of ligands or functional groups that can chelate metals [51]. Also, Zhu et al. [52] reported a higher Cu adsorption rate in manure-treated soils attributed to an increase in organic-matter-induced cation exchange capacity. However, long-term applications of manure may enrich metal levels in soil, thereby exceeding crop requirements and leading to eventual phytotoxicity [53]. In the case of Fe alteration, it might be also because cations in soils can be taken up by the BC in an anion/cation exchange and held there as an adsorption site for anions [48,51]. Regarding Cu and Fe, soil-incorporated BC can stabilize it and reduce their bioavailability through enhanced sorption and chemical precipitation [15]. As the water-soluble bioactive fraction of heavy metals in soil decreases, potential uptake and bioaccumulation of heavy metals by soil organisms (including plant roots) are minimized [54]. 

#### 4.2.4. pH and Conductivity

Conductivity and pH were affected by BC and/or manure amendment application in both studied soils. In the case of sandy loam soil, pH and conductivity increased by 9% and 94%, respectively, when manure BC was incorporated alone, although an increase (34%) and even a slight decrease (1%) were also observed when manure amendment was applied alone for conductivity and pH, respectively. pH increased by 3% in the clay loam only when manure BC was applied. Even though the conductivity was not affected in this type of soil when the studied soil amendments were applied by themselves or combined, incorporation of BC alone contributed to avoiding this parameter reduction caused by the inclusion of *C. dactylon*. 

In sandy loam soil, both BC and manure amendment altered (increased) conductivity. In general, conductivity increases as clay content increases [55]. This agrees with our study, in which 10 times more conductivity was observed in clay loam soil control pots than in the sandy loam soil ones. The high conductivity value of the clay loam soil mainly explains the lack of this parameter alteration when BC and/or manure amendment were applied. The neutral (clay loam soil) or the slight decreasing (sandy loam soil) effect of dairy manure amendment on pH did not agree with Eghball et al. [56], who reported a pH increase when manure or compost was incorporated into the top 10 cm in a clay loam soil based on *Zea mays* N and P removal; however, there is an inconsistent relationship between manure and the soil pH [57]. Hays et al. [33] also reported a neutral effect on pH when dairy manure was applied on sandy and clay loam soil in a field trial.

Although BC can increase soil cation exchange capacity (CEC) and pH [58], it might be possible that the alkaline nature of the tested BC (Table 1) was the cause of the slight pH increase in both studied soils. However, because of the similarity between the controls (sandy loam soil, pH 7.73; clay loam soil, pH 7.52) and the highest values (sandy loam + manure BC, pH 8.40; clay loam soil + manure BC, pH 7.75), it may be that these differences were too slight to have an impact in soil and plant parameters. This result partially agreed with Hays et al. [33], who also reported a slight pH increase in sandy loam and clay loam soil fields, respectively, when BC was applied in a field trial where *C. dactylon* was growing. 

## 5. Conclusions

The first notable conclusion is that the application of both manure- or wood-derived BC showed no immediate or short-term negative or positive impact on *C. dactylon* production or nutritive value in either studied soil. However, the application of dairy manure alone showed an increase in total herbage dry weight yield and N% when *C. dactylon* was grown in clay loam and sandy loam, respectively.

Considering that NO_3_-N in sandy loam soil was only increased by the incorporation of manure BC in combination with dairy manure and that P soil content was only increased when manure BC was applied (regardless of whether combined or not with manure amendment application), another relevant conclusion is that wood BC application should be considered to avoid NO_3_-N and P soil buildup when dairy manure is used as an amendment for nutrient recycling on farm.

Another consideration is that incorporation of BC improves soil total carbon content and thus could be implemented as a strategy for long-term soil C sequestration. Although both soils showed alterations in certain parameters, a net neutral (biochar) or positive (dairy manure amendment) impact on *C. dactylon* performance was observed in the short term. However, long-term field trials should be undertaken to obtain information about not only plant performance but also soil parameter alterations after subsequent application of the studied soil amendments.

## Figures and Tables

**Table 1:** Soil and manure plant available characteristic averages.

Chemical Characteristics	Sandy Loam	Clay Loam	Dairy Manure
pH	7.85	7.33	5.80
Conductivity(umhos/cm)	166	2643	6530
Oxidizable C (ppm)	198.68	1172.19	N/A
NO_3_-N (ppm)	10.86	329.52	>400.00
P (ppm)	34.77	320.35	1015.00
K (ppm)	198.18	1965.25	1070.00
Ca (ppm)	1820.53	15,967.13	2668.00
Na (ppm)	47.04	262.24	380.00
Mg (ppm)	176.04	160.00	2052.00
S (ppm)	26.38	7.83	130.00
Fe (ppm)	3.44	13.02	N/A
Zn (ppm)	0.89	4.61	N/A
Mn (ppm)	4.31	11.09	N/A
Cu (ppm)	0.36	0.72	N/A
Organic matter	N/A	N/A	71.35%
Total C	3.23%	8.65%	N/A
Total N	0.31%	0.67%	N/A

**Table 2:** Initial biochar (BC) characterization.

	Wood BC	Manure BC
	%	
Nitrogen	0.211	0.738
Phosphorus	0.004	1.149
Potassium	0.214	4.392
Calcium	0.216	6.389
Magnesium	0.035	2.615
Sodium	0.059	0.742
Ash	5.83	40.05
Fixed Carbon	60.70	23.83
Volatile Matter	27.84	32.57
	ppm	
Zinc	36.61	285.93
Iron	775.36	7708.70
Copper	12.62	153.70
Manganese	139.14	432.47
Sulfur	13.70	3167.22
Boron	2.32	29.74
		
pH	8.8	10.2

**Table 3:** Treatment combinations of biochar (BC) application and type, manure application and plant application for both studied soils.

Number	BC Application (Soil %)	BC Type	Manure Application (Mg DM/ha)	*C. dactylon*
(1) Control	0	--	0	−
(2) Control	0	--	0	+
(3)	2	Wood	0	−
(4)	2	Wood	0	+
(5)	2	Wood	10	−
(6)	2	Wood	10	+
(7)	2	Manure	0	−
(8)	2	Manure	0	+
(9)	2	Manure	10	−
(10)	2	Manure	10	+
(11)	0	--	10	−
(12)	0	--	10	+

**Table 4:** *Cynodon dactylon* second herbage cut quality (N% H-DW2) growing in a sandy loam soil and total herbage dry weight (g TH-DW) growing in a clay loam soil with Tukey’s test displaying the mean ± standard error; ANOVA one-way interaction: Manure application (SL: *p* = 0.027; CL: *p* = 0.025). Values followed by the same letter do not differ (*p* ≤ 0.05).

Manure Application	Sandy Loam H-DW2 (N%)	Clay Loam TH-DW (g)
NO	0.76 ± 0.03 b	37.7 ± 1.51 b
YES	1.18 ± 0.15 a	48.9 ± 4.55 a

**Table 5:** Sandy loam total and oxidizable C, nitrates (NO_3_-N), P and sodium (Na) and clay loam total C and N alteration with Tukey’s test displaying the mean ± standard error; ANOVA one-way interaction. Different letters within each nutrient and factor indicate least significant differences (*p* ≤ 0.05).

	Sandy Loam					Clay Loam	
	%		ppm			%	
Factor	Total C	Oxidizable C	NO_3_-N	P	Na	Total C	Total N
Plant application							
NO	3.72 ± 0.27 a	284.3 ± 45.13 a	22.2 ± 4.01 a	69.8 ± 10.34 a	82.97 ± 12.43 a	10.15 ± 0.45 a	0.68 ± 0.03 a
YES	3.48 ± 0.26 a	282.8 ± 30.13 a	1.4 ± 0.22 b	53.3 ± 8.94 b	87.73 ± 11.90 a	10.27 ± 0.31 a	0.62 ± 0.03 a
*p*-value			<0.001	<0.001			
Manure application							
NO	3.89 ± 0.32 a	198.4 ± 27.77 b	6.87 ± 1.82 a	57.25 ± 10.48 a	73.7 ± 9.92 b	9.68 ± 0.37 b	0.60 ± 0.03 b
YES	3.85 ± 0.27 a	368.7 ± 41.78 a	16.76 ± 4.48 a	66.00 ± 9.01 a	96.9 ± 13.65 a	10.75 ± 0.37 a	0.70 ± 0.03 a
*p*-value		<0.001			0.007	0.027	0.017
Biochar application (%)							
0	2.90 ± 0.10 b	298.2 ± 37.39 a	13.59 ± 4.08 a	38.14 ± 2.96 a	57.02 ± 3.61 a	9.14 ± 0.27 b	0.62 ± 0.03 a
2	3.89 ± 0.19 a	268.9 ± 39.66 a	10.05 ± 2.93 a	85.11 ± 11.66 a	113.68 ± 14.70 a	11.29 ± 0.36 a	0.67 ± 0.03 a
*p*-value	<0.001					<0.001	

**Table 6:** Sandy loam potassium (K), sodium (Na) and nitrate (NO_3_-N) as affected by biochar (BC) type and BC, manure and plant application with Tukey’s test displaying the mean ± standard error; ANOVA two-way interaction *(p* ≤ 0.05): (**a**) BC type × Manure application; (**b**) BC type × BC application; (**c**) BC application × Plant application.

(**a**)			
**Nutrient**		**Manure application**	
	**BC type**	**NO**	**YES**
NO_3_-N (ppm)	Manure	5.7 ± 2.34 b A *	23.7 ± 7.87 a A
(*p* = 0.035)	Wood	7.9 ± 2.87 a A	9.7 ± 3.66 a A
(**b**)			
**Nutrient**		**BC application (%)**	
	**BC type**	**0**	**2**
K (ppm)	Manure	155.8 ± 13.70 b A *	817.4 ± 54.52 a A
(*p* < 0.001)	Wood	161.2 ± 13.80 a A	173.5 ± 10.89 a B
Na (ppm)	Manure	57.8 ± 5.66 b A	171.6 ± 16.46 a A
(*p* < 0.001)	Wood	56.1 ± 3.55 a A	55.7 ± 4.72 a B
(**c**)			
**Nutrient**		**Plant application**	
	**BC application (%)**	**NO**	**YES**
K (ppm)	0	200.1 ± 5.4 a B *	117.0 ± 5.97 b B
(*p* = 0.01)	2	547.4 ± 110.9 a A	443.5 ± 95.63 b A

* For each nutrient, values within each column (upper case) and each line (lower case) followed by the same letter do not differ (*p* ≤ 0.05).

**Table 7:** Clay loam total N alteration with Tukey’s test displaying the mean ± standard error; ANOVA three-way interaction: Biochar (BC) type × BC application × Plant application (*p* ≤ 0.05).

		Plant Application	
Nutrient	BC Type (BC Application%)	NO	YES
Total N (%)	Manure (0)	0.77 ± 0.08 a A *	0.49 ± 0.05 a B
(*p* = 0.005)	Manure (2)	0.62 ± 0.05 a A	0.81 ± 0.05 a A
	Wood (0)	0.64 ± 0.06 a A	0.59 ± 0.03 a AB
	Wood (2)	0.69 ± 0.07 a A	0.59 ± 0.07 a AB

* Values within each column (upper case) and each line (lower case) followed by the same letter do not differ (*p* ≤ 0.05).

**Table 8:** Sandy loam P as affected by biochar (BC) type and application with Tukey’s test displaying the mean ± standard error; ANOVA three-way interaction: BC type × BC application × Manure application (*p* ≤ 0.05).

		BC Application (%)	
Nutrient	BC Type (Manure Application)	0	2
	Manure (NO)	29.9 ± 3.25 b A *	138.7 ± 15.07 a A
P (ppm)	Manure (YES)	49.5 ± 9.35 b B	128.9 ± 17.17 a A
(*p* = 0.021)	Wood (NO)	33.5 ± 1.23 a A	26.7 ± 2.67 a B
	Wood (YES)	39.6 ± 4.25 a A	45.9 ± 5.40 a B

* Values within each column (upper case) and each line (lower case) followed by the same letter do not differ (*p* ≤ 0.05).

**Table 9:** Clay loam potassium (K) alteration with Tukey’s test displaying the mean ± standard error; ANOVA two-way interaction: Biochar (BC) application × Plant application (*p* ≤ 0.05).

		Plant Application	
Nutrient	BC Application (%)	NO	YES
K (ppm)	0	1748.4 ± 170.1 a A *	893.7 ± 130.5 b A
(*p* = 0.035)	2	1588.0 ± 168.5 a A	1444.1 ± 200.0 a A

* Values within each column (upper case) and each line (lower case) followed by the same letter do not differ (*p* ≤ 0.05).

**Table 10:** Clay loam sodium (Na) alteration with Tukey’s test displaying the mean ± standard error; ANOVA three-way interaction: Biochar (BC) type × BC application × Manure application (*p* ≤ 0.05).

		BC Application (%)	
Nutrient	BC Type (Manure Application)	0	2
	Manure (NO)	223.5 ± 40.92 a A *	235.7 ± 30.11 a A
Na (ppm)	Manure (YES)	127.2 ± 29.31 b A	287.7 ± 42.65 a A
(*p* = 0.048)	Wood (NO)	178.3 ± 34.30 a A	201.4 ± 29.20 a A
	Wood (YES)	182.1 ± 24.58 a A	153.8 ± 24.78 a A

* Values within each column (upper case) and each line (lower case) followed by the same letter do not differ (*p* ≤ 0.05) according to least significant difference multiple mean separation.

**Table 11:** Sandy loam magnesium (Mg) and manganese (Mn) and clay loam sulfur (S) as affected by plant and manure application with Tukey’s test displaying the mean ± standard error; ANOVA one-way interaction (*p* ≤ 0.05). Different letters within each nutrient and factor indicate least significant differences (*p* ≤ 0.05).

	Sandy Loam		Clay Loam
		ppm	
Factor	Mg	Mn	S
Plant application			
NO	233.2 ± 16.14 a	4.23 ± 0.16 a	103.1 ± 8.66 a
YES	238.2 ± 14.64 a	3.62 ± 0.17 b	74.9 ± 7.05 b
*p*-value		0.023	0.02
Manure application			
NO	214.5 ± 15.10 b	4.12 ± 0.15 a	93.60 ± 9.05 a
YES	257.0 ± 14.43 a	3.74 ± 0.19 a	84.48 ± 7.63 a
*p*-value	0.001		

**Table 12:** Sandy loam magnesium (Mg) alteration as affected by biochar (BC) type and application rate with Tukey’s test displaying the mean ± standard error; ANOVA two-way interaction (*p* ≤ 0.05): BC type × BC application.

		BC Application (%)	
Nutrient	BC Type	0	2
Mg (ppm)	Manure	209.0 ± 12.94 b A *	337.2 ± 18.58 a A
(*p* < 0.001)	Wood	204.4 ± 10.42 a A	192.3 ± 10.20 a B

* For each nutrient, values within each column (upper case) and each line (lower case) followed by the same letter do not differ (*p* ≤ 0.05).

**Table 13:** Sandy loam copper (Cu) and iron (Fe) alteration as affected by plant, manure and biochar (BC) application with Tukey’s test displaying the mean ± standard error; ANOVA one-way interaction (*p* ≤ 0.05). Different letters within each nutrient and factor indicate differences (*p* ≤ 0.05).

	Soil Metal	
	ppm	
Factor	Cu	Fe
Plant application		
NO	0.30 ± 0.01 a	2.88 ± 0.12 a
YES	0.35 ± 0.04 a	3.67 ± 0.18 b
*p*-value		<0.001
Manure application		
NO	0.37 ± 0.04 a	3.61 ± 0.16 a
YES	0.28 ± 0.01 b	2.94 ± 0.16 b
*p*-value	0.033	<0.001
BC application (%)		
0	0.36 ± 0.04 a	3.61 ± 0.20 a
2	0.29 ± 0.01 a	2.93 ± 0.11 b
*p*-value		0.001

**Table 14:** Sandy loam pH and conductivity as affected by plant and manure application with Tukey’s test displaying the mean ± standard error; ANOVA one-way interaction (*p* ≤ 0.05). Different letters within each soil parameter and factor indicate differences (*p* ≤ 0.05).

	Soil Parameter	
Factor	pH	Conductivity (umhos/cm)
Plant application		
NO	7.92 ± 0.06 b	266.2 ± 27.45 a
YES	8.00 ± 0.05 a	210.0 ± 21.70 b
*p*-value	0.029	0.008
Manure application		
NO	8.01 ± 0.05 a	203.7 ± 20.13 b
YES	7.91 ± 0.06 b	272.5 ± 28.01 a
*p*-value	0.003	0.002

**Table 15:** Sandy loam pH and conductivity as affected by biochar (BC) type and BC application with Tukey’s test displaying the mean ± standard error; ANOVA two-way interaction: Biochar (BC) type × BC application (*p* ≤ 0.05).

		BC Application (%)	
Soil Parameter	BC Type	0	2
pH	Manure	7.73 ± 0.05 b A *	8.40 ± 0.03 a A
(*p* < 0.001)	Wood	7.80 ± 0.03 a A	7.90 ± 0.03 a B
Conductivity (umhos/cm)	Manure	196.0 ± 25.81 b A	379.5 ± 41.36 b A
(*p* = 0.001)	Wood	184.3 ± 17.08 a A	192.8 ± 17.60 a B

* For each soil parameter, values within each column (upper case) and each line (lower case) followed by the same letter do not differ (*p* ≤ 0.05).

**Table 16:** Clay loam pH alteration with Tukey’s test displaying the mean ± standard error; ANOVA three-way interaction: Biochar (BC) type × BC application × Manure application (*p* ≤ 0.05).

		BC Application (%)	
Nutrient	BC Type (Manure Application)	0	2
	Manure (NO)	7.52 ± 0.08 b AB *	7.75 ± 0.06 a A
pH	Manure (YES)	7.66 ± 0.06 a A	7.63 ± 0.06 a AB
(*p* = 0.044)	Wood (NO)	7.48 ± 0.08 a B	7.56 ± 0.08 a B
	Wood (YES)	7.58 ± 0.07 a AB	7.61 ± 0.05 a AB

* Values within each column (upper case) and each line (lower case) followed by the same letter do not differ (*p* ≤ 0.05).

**Table 17:** Clay loam conductivity alteration with Tukey’s test displaying the mean ± standard error; ANOVA two-way interaction: BC application × Plant application (*p* ≤ 0.05).

		Plant Application	
Nutrient	BC Application (%)	NO	YES
Conductivity (umhos/cm)	0	2261.0 ± 255.0 a A *	886.8 ± 90.0 b A
(*p* < 0.002)	2	1571.0 ± 240.4 a A	1280.0 ± 128.5 a A

* Values within each column (upper case) and each line (lower case) followed by the same letter do not differ (*p* ≤ 0.05).

## Data Availability

Data is contained within the article.

## References

[1] Wilkinson T.J. (1982). The definition of ancient manured zones by means of extensive sherd-sampling techniques. J. Field Archaeol..

[2] Artiola J.F., Wardell L. (2017). Guide to Making and Using Biochar for Gardens in Southern Arizona.

[3] Uzoma K.C., Inoue M., Andry H., Fujimaki H., Zahoor A., Nishihara E. (2011). Effect of cow manure biochar on maize productivity under sandy soil condition. Soil Use Manag..

[4] Zhang Z., Dong X., Wang S., Pu X. (2020). Benefits of organic manure combined with biochar amendments to cotton root growth and yield under continuous cropping systems in Xinjiang, China. Sci. Rep..

[5] United States Department of Agriculture, National Agricultural Statistic Services United States Cattle Inventory, Last Revised January 2022. https://www.nass.usda.gov/Newsroom/printable/2022/01-31-2022.pdf.

[6] United States Department of Agriculture, Natural Resources Conservation Service (1995). Animal Manure Management.

[7] Correl D.L. (1998). The Role of Phosphorus in the Eutrophication of Receiving Waters: A Review. J. Environ. Qual..

[8] Lehmann J. (2007). Bio-energy in the black. Front. Ecol. Environ..

[9] Mukherjee A., Zimmerman A., Harris W. (2011). Surface chemistry variations among a series of laboratory-produced biochars. Geoderma.

[10] Kizito S., Luo H., Lu J., Bah H., Dong R., Wu S. (2019). Role of Nutrient-Enriched Biochar as a Soil Amendment during Maize Growth: Exploring Practical Alternatives to Recycle Agricultural Residuals and to Reduce Chemical Fertilizer Demand. Sustainability.

[11] Deng Y., Zhang T., Wang Q. (2017). Biochar adsorption treatment for typical pollutants removal in livestock wastewater: A review. Eng. Appl. Biochar.

[12] Mandal A., Singh N., Purakayastha T.J. (2017). Characterization of pesticide sorption behaviour of slow pyrolysis biochars as low cost adsorbent for atrazine and imidacloprid removal. Sci. Total Environ..

[13] Peng L., Ren Y., Gu J., Qin P., Zeng Q., Shao J., Lei M., Chai L. (2014). Iron improving bio-char derived from microalgae on removal of tetracycline from aqueous system. Environ. Pillution Res..

[14] Choi Y.-K., Choi T.-R., Gurav R., Bhatia S.K., Parka Y.-L., Kima H.-J., Kan E., Yang Y.-H. (2020). Adsorption behavior of tetracycline onto *Spirulina* sp. (microalgae)-derived biochars produced at different temperatures. Sci. Total Environ..

[15] Guo M., Song W., Tian J. (2020). Biochar-facilitated soil remediation: Mechanisms and efficacy variations. Front. Environ. Sci..

[16] Woolf D., Amonette J.E., Street-Perrott F.A., Lehmann J., Joseph S. (2010). Sustainable biochar to mitigate global climate change. Nat. Commun..

[17] Kapoor A., Sharma R., Kuma A., Sepehya S. (2022). Biochar as a means to improve soil fertility and crop productivity: A review. J. Plant Nutr..

[18] Joseph S., Cowie A.L., Van Zwieten L., Bolan N., Budai A., Buss W., Cayuela M.L., Graber E.R., Ippolito J.A., Kuzyakov Y. (2021). How biochar works, and when it doesn’t: A review of mechanisms controlling soil and plant responses to biochar. GCB-Bioenergy.

[19] Sun H., Chen Y., Yi Z. (2022). After-effects of hydrochar amendment on water spinach production, N leaching, and N_2_O emission from a vegetable soil under varying N-inputs. Plants.

[20] Yi Z., Jeyakumar P., Yin C., Sun H. (2023). Effects of biochar in combination with varied N inputs on grain yield, N uptake, NH3 volatilization, and N_2_O emission in paddy soil. Front. Microbiol..

[21] Zhang H., Schroder J., He Z., Zhang H. (2014). Animal Manure Production and Utilization in the US. Applied Manure and Nutrient Chemistry for Sustainable Agriculture and Environment 2014.

[22] Artiola J.F., Rasmussen C., Freitas R. (2012). Effects of a biochar-amended alkaline soil on the growth of romaine lettuce and bermudagrass. Soil Sci..

[23] Niraula S., Choi Y.K., Payne K., Muir J.P., Kan E., Chang W.S. (2021). Dairy Effluent-Saturated Biochar Alters Microbial Communities and Enhances Bermudagrass Growth and Soil Fertility. Agronomy.

[24] Nystrom E.T. (2015). Biochar Effect on Soil Physical and Chemical Properties and Bermudagrass Growth. Master’s Thesis.

[25] Helton T.J., Butler T.J., McFarland M.L., Hons F.M., Mukhtar S., Muir J.P. (2008). Effects of Dairy Manure Compost and Supplemental Inorganic Fertilizer on Coastal Bermudagrass. Agron. J..

[26] Muir J.P., Butler T., Helton T.J., McFarland M.L. (2010). Dairy Manure Compost Application Rate and Timing Influence Bermudagrass Yield and Nutrient Concentration. Crop Sci..

[27] Mosebi P.E., Truter W.F., Madakadze I.C. (2015). Manure from cattle as fertilizer for soil fertility and growth characteristics of Tall Fescue (*Festuca arundinacea*) and Smuts Finger grass (*Digitaria eriantha*). Livest. Res. Rural. Dev..

[28] Cooper C.P. (2023). Short-Term Effects of Biochar on Forage Nutrient Concentrations. Master’s Thesis.

[29] Adekiyaa A.O., Agbedeb T.M., Aboyejia C.M., Dunsina O., Simeona V.T. (2019). Effects of biochar and poultry manure on soil characteristics and the yield of Radish. Sci. Hortic..

[30] Major J., Rondon M., Molina D., Riha S.J., Lehmann J. (2010). Maize yield and nutrition during 4 years after biochar application to a Colombian savanna oxisol. Plant Soil.

[31] El-Shony M.A.M., Farid I.M., El-Kamar F.A., Abbas M.H.H., Abbas H.H. (2019). Ameliorating a Sandy Soil Using Biochar and Compost Amendments and Their Implications as Slow Release Fertilizers on Plant Growth. Egypt. J. Sci..

[32] Kuziemska B., Trebicka J., Wysokinski A., Jaremko D. (2021). Supplementation of Organic Amendments Improve Yield and Adaptability by Reducing the Toxic Effect of Copper in Cocksfoot Grass (*Dactylis glomerata* L. Cv Amera). Agronomy.

[33] Hays K.N., Muir J.P., Kan E., DeLaune P.B., Brady J.A., Obayomi O., Mitchell A.B. (2023). Tillage, Manure, and Biochar Short-Term Effects on Soil Characteristics in Forage Systems. Agronomy.

[34] Gross A., Glaser B. (2021). Meta-analysis on how manure application changes soil organic carbon storage. Sci. Rep..

[35] Chen Z., Liu J., Sun H., Xing J., Zhang Z., Jiang J. (2023). Effects of Biochar Applied in Either Rice or Wheat Seasons on the Production and Quality of Wheat and Nutrient Status in Paddy Profiles. Plants.

[36] Taggart C.B., Muir J.P., Brady J.A., Kan E., Mitchell A.B., Obayomi O. (2023). Impacts of Biochar on *Trifolium incarnatum* and *Lolium multiflorum*: Soil Nutrient Retention and Loss in Sandy Loam Amended with Dairy Manure. Agronomy.

[37] Sanderson M.A., Jones R.M. (1997). Forage yields, nutrient uptake, soil chemical changes, and nitrogen volatilization from bermudagrass treated with dairy manure. J. Prod. Agric..

[38] Culman S., Freeman M., Snapp S. (2014). Procedure for the Determination of Permanganate Oxidizable Carbon.

[39] Mehlich A. (1978). New extractant for soil test evaluation of phosphorus, potassium, magnesium, calcium, sodium, manganese, and zinc. Commun. Soil Sci. Plant Anal..

[40] Liu M., Che Y., Wang L., Zhao Z., Zhang Y., Wei L., Xiao Y. (2019). Rice straw biochar and phosphorus inputs have more positive effects on the yield and nutrient uptake of Lolium multiflorum than arbuscular mycorrhizal fungi in acidic Cd-contaminated soils. Chemosphere.

[41] Demiraj E., Libutti A., Malltezi J., Rroco E., Brahushi F., Monteleone M., Sulce S. (2018). Effect of organic amendments on nitrate leaching mitigation in a sandy loam soil of Shkodra district, Albania. Ital. J. Agron..

[42] Demisie W., Liua Z., Zhang M. (2014). Effect of biochar on carbon fractions and enzyme activity of red soil. Catena.

[43] Angers D.A., N’Dayegamiye A. (1991). Effects of manure application on carbon, nitrogen, and carbohydrate contents of a silt loam and its particle-size fractions. Biol. Fertil. Soils.

[44] Demirel B., Yenigun O., Onay T.T. (2005). Anaerobic treatment of dairy wastewaters: A review. Process Biochem..

[45] Hagemann N., Kammann C.I., Schmidt H.P., Kappler A., Behrens S. (2017). Nitrate capture and slow release in biochar amended compost and soil. PLoS ONE.

[46] Steiner C., Das K.C., Melear N., Lakly D. (2010). Reducing nitrogen loss during poultry litter composting using biochar. J. Environ. Qual..

[47] Biederman L.A., Harpole W.S. (2013). Biochar and its effects on plant productivity and nutrient cycling: A meta-analysis. GCB Bioenergy.

[48] Zhao S., Wang B., Gao Q., Gao Y., Liu S. (2017). Adsorption of Phosphorus by Different Biochars. Spectrosc. Lett..

[49] Glaser B., Lehmann J., Zech W. (2002). Ameliorating physical and chemical properties of highly weathered soils in the tropics with charcoal–A review. Biol. Fertil. Soils.

[50] Atkinson C.J., Fitzgerald J.D., Hipps N.A. (2010). Potential mechanisms for achieving agricultural benefits from biochar application to temperate soils: A review. Plan Soil.

[51] Harter R.D.R., Naidu R. (1995). Role of metal-organic complexation in metal sorption by soils. Adv. Agron..

[52] Zhu Y.M., Berry D.F., Martens D.C. (1991). Copper availability in two soils amended with 11 annual applications of copper-enriched hog manure. Commun. Soil Sci. Plant Anal..

[53] Bolan N., Adriano D., Mahimairaja S. (2004). Distribution and Bioavailability of Trace Elements in Livestock and Poultry Manure By-Products. Crit. Rev. Environ. Sci. Technol..

[54] Ahmad M., Rajapaksha A.U., Lim J.E., Zhang M., Bolan N., Mohan D., Vithanage M., Lee S.S., Ok Y.S. (2014). Biochar as a sorbent for contaminant management in soil and water: A review. Chemosphere.

[55] USDA Natural Resources Conservation Service (2011). Soil Quality Indicators. https://www.nrcs.usda.gov/sites/default/files/2022-10/Soil%20Electrical%20Conductivity.pdf.

[56] Eghball B., Ginting D., Gilley J.E. (2004). Residual Effects of Manure and Compost Applications on Corn Production and Soil Properties. Agron. J..

[57] Rayne N., Aula L. (2020). Livestock Manure and the Impacts on Soil Health: A Review. Soil Syst..

[58] Weil R.R., Brady N.C. (2019). Elements of the Nature and Properties of Soils.

